# Adrenal infarction with latent myelodysplastic/myeloproliferative neoplasm, unclassifiable with *JAK2* V617F mutation

**DOI:** 10.1002/ccr3.8729

**Published:** 2024-04-09

**Authors:** Shunichiro Yasuda, Momoko Chiba, Rie Nishitani, Takako Watanabe

**Affiliations:** ^1^ Department of Hematology Tokyo Kyosai Hospital Tokyo Japan; ^2^ Department of Diabetes, Endocrinology and Metabolism Tokyo Kyosai Hospital Tokyo Japan

**Keywords:** adrenal infarction, CHIP, *JAK2*V617F, myelodysplastic/myeloproliferative neoplasm, unclassifiable (MDS/MPN‐U)

## Abstract

**Key Clinical Message:**

Hematopoietic neoplasms can cause adrenal infarction. In cases of thrombosis occurring at uncommon sites, it is necessary to consider evaluating for the *JAK2*V617F mutation, even in the absence of notable abnormalities in blood counts.

**Abstract:**

Adrenal infarction, a rare ailment, has been sporadically linked to hematopoietic neoplasms. A 46‐year‐old male encountered left adrenal infarction, which coincided with a progressive rise in platelet counts. Subsequent diagnosis revealed myelodysplastic/myeloproliferative neoplasm‐unclassifiable, featuring a *JAK2*V617F mutation. Simultaneously, the patient manifested multiple arteriovenous thromboses, necessitating treatment with edoxaban, aspirin, and hydroxyurea. Following thrombosis resolution, he was transferred to a transplantation center. This report delves into the thrombogenicity linked to the *JAK2*V617F mutation, while also examining documented instances of adrenal infarction in myeloid neoplasms. We should consider evaluating for *JAK2*V617F mutation even in cases of thrombosis at unusual sites, including adrenal infarction, even if there are no considerable abnormalities in blood counts.

## 1 INTRODUCTION

Adrenal infarction is a rare condition that occasionally develops in a hypercoagulable state.^1^ Antiphospholipid antibody syndrome, COVID‐19 infection, and heparin‐induced thrombocytopenia have been reported as underlying diseases of adrenal infarction.^1^, ^2^, ^3^ Adrenal infarctions have also been rarely linked to myeloproliferative neoplasms (MPN), such as polycythemia vera (PV) and essential thrombocythemia (ET).^4^, ^5^

Myelodysplastic/myeloproliferative neoplasm (MDS/MPN) is a hematological disorder that exhibits characteristics of both MDS and MPN.^6^, ^7^ According to the revised version of the fourth edition of the World Health Organization (WHO) classification, the categories of MDS/MPN include chronic myelomonocytic leukemia, juvenile myelomonocytic leukemia, atypical chronic myeloid leukemia, MDS/MPN with ring sideroblasts and thrombocytosis (MDS/MPN‐RS‐T), and MDS/MPN‐unclassifiable (MDS/MPN‐U).^6^, ^7^, ^8^ The diagnosis of MDS/MPN‐U is based on ruling out any other subtype of MDS/MPN.^7^, ^8^ Approximately 25% of cases with MDS/MPN‐U have *JAK2*V617F mutations,^8^ which is a major mutation in patients with MPN.^9^ As patients with MDS/MPN‐U exhibit MPN characteristics, they may have a high risk of thrombosis. However, due to the rarity of this disease, the frequency of thrombosis in patients with MDS/MPN‐U has not yet been thoroughly investigated.

Herein, we present a case of *JAK2*V617F‐positive MDS/MPN‐U with adrenal infarction. The present case had a unique clinical course in which the platelet count of the patient was almost normal at the time of adrenal infarction; however, later, the counts increased, and the characteristics of MPN became apparent. We report this case with a discussion of the thrombogenicity caused by the *JAK2*V617F mutation and review previously reported cases of adrenal infarction in myeloid neoplasms.

## 2 CASE HISTORY/EXAMINATION

**FIGURE 1:**
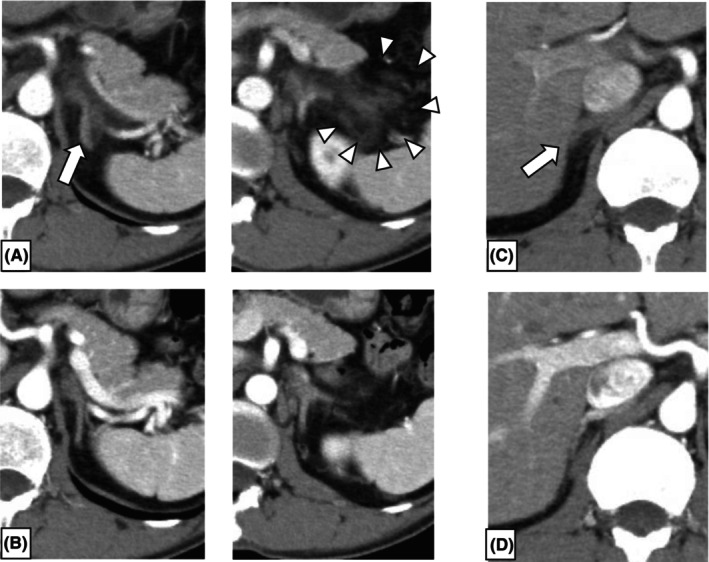
Contrast‐enhanced computed tomography (CT) showing development (A) and improvement of (B) the left adrenal infarction. Contrast‐enhanced CT showing development (C) and improvement (D) of the right adrenal infarction. (A) The arrow indicates hypertrophy of the left adrenal gland and non‐contrast‐enhanced areas, and arrowheads denote increased lipid concentrations and fluid retention around the left adrenal gland. The patient was diagnosed with left adrenal infarction. (B) Improvement of the left adrenal infarction. (C) The arrow indicates mild hypertrophy of the right adrenal gland. The patient was diagnosed with right adrenal infarction. (D) Improvement of the right adrenal infarction.

**FIGURE 2:**
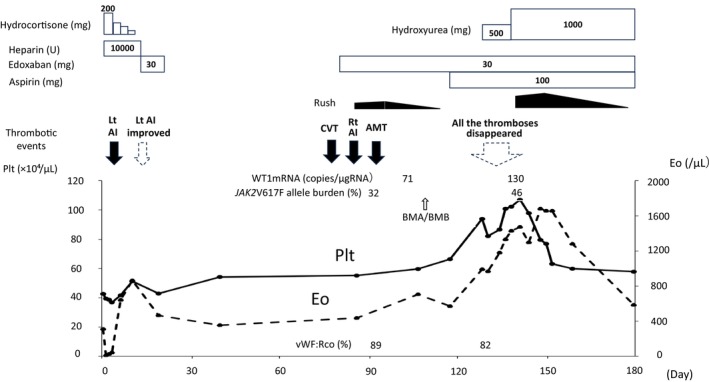
The clinical course of the adrenal infarction with *JAK2*V617F‐positive MDS/MPN‐U with der(1;7)(p10;q10). AI, adrenal infarction; AMT, aortic mural thrombosis; BMA, bone marrow aspiration; BMB, bone marrow biopsy; CVT, cerebral venous thrombosis; Eo, eosinophil; Lt, left; Plt, platelet; Rt, right; U, unit; vWF:Rco, von Willebrand factor to ristocetin cofactor activity.

A 46‐year‐old previously healthy man presented to our emergency department with epigastric and left hypochondrial pain. He had no history of medication or family history of thrombophilia. On physical examination, his body temperature was 37.1°C, blood pressure was 152/101 mmHg, heart rate was 118 bpm, and oxygen saturation was 97%. Electrocardiography findings were normal, and chest radiograph showed no abnormalities. Blood tests revealed a white blood cell (WBC) count of 9.7 × 10^3^/μL, a hemoglobin level of 12.9 g/dL with a mean corpuscular volume of 104.7 fL, and a platelet count of 42.6 × 10^4^/μL. The differential showed 81% neutrophils with an absolute neutrophil count of 7.9 × 10^3^/μL, 3% monocytes with an absolute monocyte count of 0.3 × 10^3^/μL, and 3% eosinophils with an absolute eosinophil count of 0.3 × 10^3^/μL. The COVID‐19 antigen test was negative. Contrast‐enhanced computed tomography (CT) showed left adrenal hypertrophy and non‐contrast‐enhancing areas of the adrenal gland. Additionally, there was increased density of adipose tissue and fluid retention around the left adrenal gland (Figure 1A). Subsequently, he received a diagnosis of left adrenal infarction. The patient was urgently admitted to our hospital, and heparin 10,000 U/day was initiated (Figure 2). While the adrenal infarction was unilateral, there were no signs of adrenal insufficiency based on vital signs and laboratory data.

## 3 DIFFERENTIAL DIAGNOSIS, INVESTIGATIONS, AND TREATMENT

Due to the potential for adrenal insufficiency, we initiated the administration of hydrocortisone as a steroid cover. Endocrine examinations indicated the maintenance of the adrenal function of the patient; consequently, the hydrocortisone dose was tapered and terminated. His epigastric and left hypochondrial pain steadily relieved, and follow‐up contrast‐enhanced CT on Day 7 revealed resolution of the left adrenal infarction (Figure 1B). Subsequently, we transitioned the antithrombotic therapy from heparin 10,000 U/day to edoxaban 30 mg/day, and the patient was discharged on Day 16. As for the thrombotic predisposition that led to the adrenal infarction, the increase in platelet counts was not remarkable. Both protein C activity and protein S activities were within the normal range (148% and 73%, respectively). The lupus anticoagulant was negative. He did not experience atrial fibrillation during his hospitalization. Hence, we could not find any causes of adrenal infarction.

## 4 OUTCOME AND FOLLOW‐UP

Soon after discharge from our hospital, the patient suffered from a headache. Considering the possibility of edoxaban‐induced headache, edoxaban treatment was discontinued on Day 20. Two months later, he presented to our hospital with a persistent headache despite discontinuing edoxaban treatment. He was diagnosed with cerebral venous thrombosis (CVT) on contrast‐enhanced magnetic resonance imaging (MRI), and edoxaban treatment was resumed on Day 88. Simultaneously, the patient presented with right hypochondrial pain, and contrast‐enhanced CT showed mild hypertrophy of the right adrenal gland (Figure 1C). He was clinically diagnosed with right adrenal infarction. Furthermore, abdominal aortic mural thrombosis was incidentally detected on contrast‐enhanced CT. Regarding multiple arteriovenous thromboses in a short period, his thrombotic predisposition was examined again. Blood tests showed that various autoantibodies were all negative, and the patient did not have diabetes or dyslipidemia. Blood tests revealed a WBC count of 8.7 × 10^3^/μL, and the differential indicated 63.2% neutrophils, 3.7% monocytes, and 8.1% eosinophils (Table 1). However, the platelet counts, which were almost normal at the time of the first admission, gradually increased (Figure 2). Suspecting MPN, especially ET, we examined the presence of *JAK2*V617F mutation, which was found to be positive. Bone marrow aspiration showed no increase in the blasts but showed dysplastic features in granulocytes, erythroblasts, and megakaryocytes; a chromosomal abnormality with der(1;7)(q10;p10) was found in 20 out of 20 cells. Although our patient had mild eosinophilia, he tested negative for both FIP1L1‐PDGFRA and FGFR1 using fluorescence in situ hybridization. Bone marrow biopsy showed no bone marrow fibrosis. WT1mRNA was slightly high (71 copies/μgRNA). As our patient had characteristics of both MDS and MPN and the diagnostic criteria for other MDS/MPN subtypes were not fulfilled, he was diagnosed with MDS/MPN‐U. At the same time, the patient was aware of a rash resembling urticaria rash and pruritus on both lower legs, with mild increase in eosinophils (8.1%). The rash and pruritus improved with the use of an antihistamine cream. As his thrombocytosis was not remarkable (59.7 × 10^4^/μL) and von Willebrand factor (vWF) ristocetin cofactor activity (vWF:RCo) did not decrease (89%), the risk of bleeding might not be high. The patient was then treated with edoxaban (30 mg/day) and aspirin (100 mg/day).

**TABLE 1:** Laboratory data at the time of recurrent thrombotic events, including CVT, right adrenal infarction, and AMT.

Complete blood count		Blood chemistry		Immunologic test	
WBC	8700/μL	BUN	12 mg/dL	IgG	816 mg/dL
Neut ANC	63.2% 5498/μL	Cre	0.92 mg/dL	IgA	139 mg/dL
Lymph	21.5	AST	45 U/L	IgM	82 mg/dL
Mono AMC	3.7% 322/μL	ALT	21 U/L	C3	97 mg/dL
Eo AEC	8.1% 705/μL	LDH	357 U/L	C4	30 mg/dL
Baso	5.0	ALP	42 U/L	Antinuclear antibody	(−)
RBC	354 × 10^4^	γGTP	23 U/L	Anti‐dsDNA IgG antibody	(−)
Hb	12.4 g/dL	T.Bil	0.7 mg/dL	Lupus anticoaglant	(−)
Ht	36.3%	Na	140 mEq/L	aCL‐β2GPI	(−)
MCV	102.5 fL	K	4.8 mEq/L	Anti‐cardiolipin antibody	(−)
MCHC	34.2 g/dL	Cl	104 mEq/L	Genetic test	
Plt	59.7 × 10^4^/μL	CRP	0.04 mg/dL	*JAK2*V617F	(+)
Coagulation test		TG	46 mg/dL	allele burden	32%
PT	19.3 s	T‐Chol	135 mg/dL	*BCR*::*ABL* (FISH)	(−)
PT	55.7%	LDL‐Chol	64 mg/dL	WT1mRNA	71 Copies/μgRNA
PT‐INR	1.44	HDL‐Chol	55 mg/dL	Endocrine test	
APTT	43.5 s	Glucose	91 mg/dL	ACTH	37.3 pg/mL
Fib	309 mg/dL	HbA1c	5.2%	Cortisol	15.7 μg/dL
D‐dimer	0.92 μg/mL	Haptoglobin	49 mg/dL	DHEA‐S	347 μg/dL
Antithrombin III	112%	Vitamin B12	552 pg/mL	PRA	<0.2 ng/mL/h
vWF:RCo	89%	Folic acid	4.5 ng/mL	PAC	23 pg/mL

Abbreviations: AEC, absolute eosinophil count; AMC, absolute monocyte count; AMT, aortic mural thrombosis; ANC, absolute neutrophil count; anti‐CL β2GPI Ab, anti‐cardiolipin β2‐glycoprotein‐I complex antibody; CVT, cerebral venous thrombosis; DHEA‐S, dehydroepiandrostendione sulfate; MCV, mean corpuscular volume; PAC, plasma aldosterone concentration; PRA, plasma renin activity; vWF:Rco, von Willbrand factor to ristocetin cofactor activity; WBC, white blood cell.

On Day 131, the patient was admitted to our hospital again for close examination and treatment because his platelet counts further increased (Figure 2) and the development of a new thrombosis was expected. After admission, contrast‐enhanced MRI and contrast‐enhanced CT were performed, which showed resolution of all arteriovenous thromboses, including the right adrenal infarction (Figure 1D). The platelet counts rose to approximately 100 × 10^4^/μL; therefore, hydroxyurea 500 mg/day was started on Day 137 as cytoreductive therapy, which was increased to 1000 mg/day on Day 140. His eosinophil count also gradually increased with a skin rash flare‐up. A skin biopsy was performed, leading to the pathological diagnosis of leukocytoclastic vasculitis, which was considered closely related to MDS with der(1;7)(q10;p10).^10^, ^11^ After the hydroxyurea treatment, both the platelet and eosinophil counts decreased, leading to no recurrence of new thrombosis or improvement of the skin rash. Both *JAK2*V617F allele burden and the copy numbers of WT1mRNA increased in our patient. Furthermore, MDS with der(1;7)(p10;q10) complicated by eosinophilia has aggressive clinical features and a poor prognosis.^11^ As the prognosis of allogeneic hematopoietic stem cell transplantation (allo‐HSCT) is poor in patients with MDS/MPN‐U who have progressive disease or severe complications,^12^ earlier allo‐HSCT might be preferable. After obtaining adequate informed consent, including the risks and benefits of allo‐HSCT, the patient was transferred to the transplantation center to receive allo‐HSCT at the optimal time. At the time of submission, our patient had not yet received allo‐HSCT and was still under outpatient observation.

## 5 DISCUSSION

In this manuscript, we present a case of *JAK2*V617F‐positive MDS/MPN‐U with multiple thromboses, including adrenal infarction. Although the precise mechanisms of thrombogenesis in MDS/MPN‐U have not been elucidated, we speculate that MDS/MPN‐U caused multiple thromboses as our patient did not have any other risk factors for thrombosis or a thrombotic predisposition. Notably, the first adrenal infarction occurred when the platelet count was only slightly elevated and MDS/MPN was not apparent. To date, various mechanisms of thrombogenesis by *JAK2*V617F have been reported; (1) *JAK2*V617F‐positive neutrophils and monocytes release inflammatory cytokines, leading to arteriosclerosis and arterial thrombosis.^13^ (2) *JAK2*V617F‐positive neutrophils activate β1/2 integrin, promoting venous thrombosis.^14^, ^15^ (3) Vascular endothelial cell expression of *JAK2*V617F promotes a prothrombotic state due to increased P‐selectin expression.^16^ Recently, clonal hematopoiesis (CH) has been identified and genetic mutations associated with the disease have been detected before the development of hematological malignancies.^17^, ^18^ In addition, when CH occurs and the variant allele frequency (VAF) exceeds 2%, it is called CH with indeterminate potential (CHIP).^17^ *JAK2*V617F‐CHIP holders had a higher incidence of both arterial and venous thromboses than non‐holders.^17^ In the present case, *JAK2*V617F‐CHIP may have been associated with the thrombogenicity of adrenal infarction.

There were some cases of splanchnic thrombosis in patients with the *J*AK2V617F mutation^19^; however, there were only a few cases involving adrenal infarction. Previously, five cases of myeloid neoplasms that developed into adrenal infarctions have been reported (Table 2). Among these cases, two patients with ET had *JAK2*V617F mutations.^4^, ^5^ Indeed, one patient developed adrenal infarction before the diagnosis of ET, indicating that *JAK2*V617F‐CHIP was closely associated with adrenal infarction, as in our case.^4^ However, one patient with MDS/MPN‐U did not harbor the *JAK2*V617F mutation,^22^ indicating that other gene mutations may contribute to the thrombogenesis in MDS/MPN‐U. Furthermore, one case with MDS had adrenal infarction with no other thrombotic predisposition, such as MPN, suggesting that the hypercoagulable state associated with MDS may contribute to thrombogenesis.^20^ With reference to these case reports, other factors as well as *JAK2*V617F mutation, may have caused the adrenal infarction in our case with MDS/MPN‐U. A previous study suggested that *SF3B1* may be a risk factor for thrombosis in MDS/MPN‐RS‐T, a subtype of MDS/MPN.^23^ At present, *SF3B1* mutation analysis is required for the accurate classification of MDS/MPN subtypes according to the latest fifth WHO classification. However, *SF3B1* analysis can only be performed at a limited number of centers in Japan and is not available at our hospital. If *SF3B1* or other gene mutation analyses become widespread and can be performed in daily practice, more accurate predictions can be made regarding the risk of thrombosis in MDS/MPN subtypes in the future.

**TABLE 2:** Previous cases with myeloid neoplasms that developed adrenal infarction.

No	Age	Sex	Disease	*JAK2* V617F	Plt (×10^4^/μL)	Uni or bi adrenal infarction	Other thromboses	Adrenal insufficiency	Chemotherapy/cytoreductive therapy	Antithrombotic agents	Outcome of adrenal infarction	Reference
1	63	F	MDS	N/A	35.6	Bi	Splenic infarction	+	−	Warfarin	Progressed	[20]
2	49	M	AML	N/A	90.0	Bi	−	+	AZA	Aspirin	Improved	[21]
3	64	M	ET	+	121.7	Bi	Splenic infarction, AMT, AP	−	HU, ANA	Aspirin, prasugrel	Improved	[5]
4	76	M	ET	+	24.2	Bi	AMT	+	HU	Aspirin	Improved	[4]
5	81	M	MDS/MPN‐U	−	46.8	Bi	−	+	AZA	Aspirin	Improved	[22]
6	46	M	MDS/MPN‐U	+	42.6	Multiple Uni	CVT, AMT	−	HU	Aspirin, edoxaban	Improved	Our case

Abbreviations: Plt, platelet counts at the time of adrenal infarction; AML, acute myelogenous leukemia; AMT, aortic mural thrombosis; ANA, anagrelide; AP, angina pectoris; AZA, azacitidine; Bi, bilateral; CVT, cerebral venous thrombosis; ET, essential thrombocythemia; F, female; HU, hydroxyurea; M, male; MDS, myelodysplastic syndrome; MPN, myeloproliferative neoplasm; N/A, not applicable; U, unclassifiable; Uni, unilateral.

All six cases in Table 2, including the current case, developed complications such as adrenal insufficiency and thromboses at other sites. Among the six patients, five had bilateral adrenal infarction^4^, ^5^, ^20^, ^21^, ^22^ and four developed primary adrenal insufficiency that required steroid replacement therapies.^4^, ^20^, ^21^, ^22^ In our case, the adrenal infarction was unilateral and improved soon after edoxaban treatment. Although our patient subsequently developed adrenal infarction contralaterally, the lesions of adrenal infarction were small; therefore, the patient did not develop adrenal insufficiency.

Among the six patients shown in Table 2, four developed thromboses at other sites, including one case of angina pectoris that required percutaneous coronary intervention.^5^ Our patient simultaneously developed CVT, abdominal aortic mural thrombosis, and adrenal infarction. In accordance with the recommendations of a previous study on the combination of antiplatelet and anticoagulant drugs for arteriovenous thrombosis in patients with MPN,^24^ the patient was treated with edoxaban and aspirin, and all thromboses disappeared.

Although the six cases in Table 2 did not develop adrenal hemorrhagic infarction, they sometimes occurred after adrenal infarction. The mechanisms underlying adrenal infarction and adrenal hemorrhagic infarction are not fully understood; however, it is hypothesized that they are related to the unique vascular anatomy of the adrenal gland.^4^, ^22^, ^25^ When treating adrenal infarction, attention should be paid to adrenal hemorrhagic infarction. In particular, patients with MPN have an increased risk of bleeding owing to a decrease in vWF activity associated with an increase in the platelet count.^26^ Therefore, patients with adrenal infarction who have characteristics of MPN should be examined for vWF activity before starting treatment with antithrombotic agents.

Patients with MDS/MPN‐U generally have a poor prognosis with a median overall survival of 12.4 months, as reported by a previous study.^8^ Although the efficacy of ruxolitinib or hypomethylating agents for MDS/MPN‐U has been reported,^27^, ^28^ they are not curative, and the only curative treatment option is allo‐HSCT.^12^ As patients with severe complications before allo‐HSCT have a worse prognosis, management of these complications is crucial for patients with MDS/MPN‐U. Although our patient had multiple arteriovenous thromboses, they were managed with antithrombotic agents, and the patient was eventually transferred to the transplantation center without any complications.

## 6 CONCLUSION

We report a case of *JAK2*V617F‐positive MDS/MPN‐U with adrenal infarction. Hematopoietic neoplasms are considered a differential diagnosis when encountering adrenal infarction with unknown causes. Even if there are no abnormal findings in the blood cell counts at the time of adrenal infarction, they should be carefully monitored, and when MPN is suspected, *JAK2*V617F mutation should be examined. Finally, patients with adrenal infarction of hematopoietic neoplasms often have complications, and management of these complications is crucial, especially in patients eligible for allo‐HSCT.

## AUTHOR CONTRIBUTIONS

**Shunichiro Yasuda:** Conceptualization; methodology; project administration; writing – original draft; writing – review and editing. **Momoko Chiba:** Conceptualization; project administration; writing – review and editing. **Rie Nishitani:** Project administration; writing – original draft. **Takako Watanabe:** Funding acquisition; supervision; writing – review and editing.

## FUNDING INFROMATION

There is no funding for this article.

## CONFLICT OF INTEREST STATEMENT

The authors have no conflicts of interest relevant to the content of this article.

## ETHICS STATEMENT

This manuscript confirms to the provisions of the Declaration of Helsinki in 1995 (as revised in Brazil 2013).

## CONSENT

Written informed consent was obtained from the patient to publish this report in accordance with the journal's patient consent policy.

## Data Availability

The data that support the findings of this study are available from the corresponding author upon reasonable request.
